# Synthesis and Evaluation of Anti-acetylcholinesterase Activity of 2-(2-(4-(2-Oxo-2-phenylethyl)piperazin-1-yl) ethyl)Isoindoline-1,3-dione Derivatives with Potential Anti-Alzheimer Effects

**Published:** 2013-10

**Authors:** Alireza Aliabadi, Alireza Foroumadi, Ahmad Mohammadi-Farani, Mahdi Garmsiri Mahvar

**Affiliations:** 1 Novel Drug Delivery Research Center, Kermanshah University of Medical Sciences, Kermanshah, Iran; 2 Department of Medicinal Chemistry, Faculty of Pharmacy, Kermanshah University of Medical Sciences, Kermanshah, Iran; 3 Department of Medicinal Chemistry, Faculty of Pharmacy and Pharmaceutical Sciences Research Center, Tehran University of Medical Sciences, Tehran, Iran; 4 Department of Pharmacology, Toxicology and Medical Services, Faculty of Pharmacy, Kermanshah University of Medical Sciences, Kermanshah, Iran; 5 Students Research Committee, Kermanshah University of Medical Sciences, Kermanshah, Iran

**Keywords:** Acetylcholinesterase Alzheimer, Phthalimide, Synthesis

## Abstract

***Objective(s):*** Alzheimer's disease (AD) is a neurodegenerative disorder in elderly patients. Decrease in cholinergic neurotransmission is the main known cause in the pathophysiology of the disease. Improvement and potentiation of the cholinergic system could be beneficial for treatment of the AD. Acetylcholinesterase inhibitors such as donepezil can enhance the duration of action of acetylcholine (Ach) and therefore, through this mechanism improve the symptoms of AD. ***Materials and Methods:*** In the current study, based on the potential inhibitory activity of phthalimide derivatives towards acetylcholinesterase enzyme, a new series of phthalimide-based compounds were synthesized (**4a-4e**) and anti-acetylcholinesterase effect was assessed using Ellman's test. Compound 4b with 4-Fluorophenyl moiety was the most potent derivative in this series (IC_50_ = 16.42 ± 1.07 µM). It was shown that, none of the synthesized compounds showed superior inhibitory potency compared to donepezil (0.41 ± 0.09 µM) as a reference drug.

***Conclusion: ***The new synthesized phthalimide based analogs could function as potential acetylcholinesterase inhibitors. Further studies are necessary for development of potent analogs**.**

## Introduction

Alzheimer's disease (AD) as an age-related and neurodegenerative disease destroys patient’s memory and cognition and also affects the communication ability of the patient. AD is the most common and the most prevalent cause of dementia with ageing. It is responsible for 50% cases of dementia in geriatric patients over 65 years of age. This progressive disorder also affects the ability to perform daily activities, doing judgment, learning and etc (1-5). Decrease in the functional capacity will result in death, approximately 8-10 years after the initiation of the symptoms of the illness (6). 

**Figure 1:**
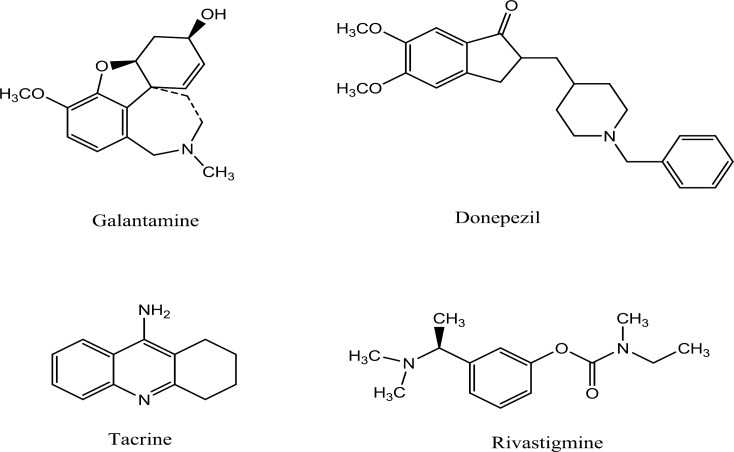
Structures of some current acetylcholinesterase inhibitors in the market

A progressive reduction in cholinergic neurons in some areas of the brain such as cortex and hippocampus is related to the deficits in memory and cognitive function in Alzheimer’s disease (AD). This observation led to the development of therapeutic agents that function as acetylcholinesterase (AChE) inhibitors in central nervous system. In fact, these agents prolong the duration of action of acetylcholine (ACh) and render symptomatic relief in this disorder (7-11). The administration of acetylcholinesterase inhibitors as first generation anti-Alzheimer drugs has beneficial effects on cognitive, functional and behavioral symptoms of the disease (2, 12). Several acetylcholinesterase inhibitors are available currently in the market such as donepezil (benzylpiperidine derivative), rivastigmine (carbamate derivative), tacrine (aminoacridine derivative) and galantamine (a natural alkaloid extracted from the herb) (13, 14) (Figure 1).

**Figure 2.**
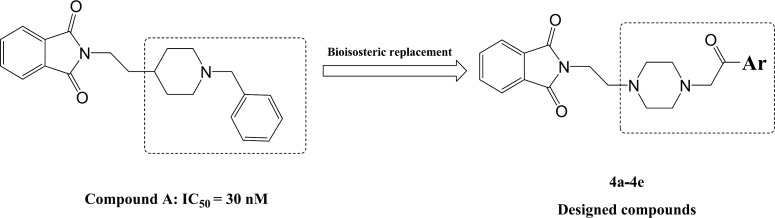
Structure of a lead compound (compound A) and the logic and process for design of new derivatives

Recently, some investigations have been reported the potential anticholinesterase activity of phthalimide derivatives (15-20). In the present study, we have focused on the structure of donepezil and therefore a new series of donepezil like derivatives based on phthalimide structure were designed as an acetylcholinesterase inhibitor with potential anti-Alzheimer effect. Furtheremore, according to the report of Sugimoto and collegues, the design of new derivatives was carried out via the bioisosteric replacement of piperidine with piperazine ring as well as benzyl moiety with various phenacyl groups (Figure 2).

## Materials and Methods

***Chemistry***

 Chemical substances, reagents and solvents were prepared from the commercial vendors such as Merck and Sigma-Aldrich companies. ^1^H-NMR spectra were recorded using a Bruker 400 MHz spectrometer in deutrated solvents, and chemical shifts are expressed as δ (ppm) with tetramethylsilane (TMS) as internal standard. The IR spectra were obtained on a Shimadzu 470 spectrophotometer using potassium bromide (KBr) disks. Melting points were determined using Electrothermal 9001 elemental analyzer apparatus and are uncorrected. The mass spectra were run on a Finigan TSQ-70 spectrometer (Finigan, USA) at 70 eV.

***Synthesis of 2-(2-(piperazin-1-yl)ethyl)isoindoline-1,3-dione*** **(3)**

 In a flask 3 g (20 mmol) of phthalic anhydride, 2.6 ml (20 mmol) *N*-amnioethylpiperazine and 2.9 ml (20 mmol) triethylamine (Et_3_N) were mixed in 40 ml of toluene solvent. The reaction mixture was refluxed for 24 hr and the termination of reaction and formation of the desired product was confirmed by thin layer chromatography. The discoloration of the reaction medium and formation of a yellow precipitate was also an indicator of the progress of the reaction. Then, toluene was evaporated under reduced pressure using rotary evaporator apparatus and the obtained yellow viscose and oily residue was washed several times by ethyl acetate (EtOAc) and diethyl ether (Et_2_O) (19).

^1^H NMR (CDCl_3_, 400 MHz) δ (ppm): 2.37 (m, Piperazine), 2.54 (m, Piperazine), 3.22 (t, phthalimide-CH_2_-CH_2_-piperazine), 3.44 (t, phthalimide-CH_2_-CH_2_-piperazine), 4.73 (s, NH, Piperazine), 7.35-7.85 (m, 4H, Phthalimide). IR (KBr, cm^-1^) ύ: 3380, 3330, 3157, 3111, 2924, 1730, 1681, 1521, 1489, 1458, 1328, 1303, 1186, 1143, 1035, 910, 750, 710. MS (*m/z*, %): 259 (M^+^, 10), 224 (30), 174 (30), 160 (60), 149 (85), 99 (100), 70 (70), 57 (65), 41 (40).

***General procedure for synthesis of compounds*** ***4a-4e***

In a flat bottom flask, equimolar quantities of compound **3**, triethylamine (Et_3_N) and appropriate derivative of arylacyl chloride were mixed in acetonitrile (CH_3_CN) solvent. The reaction was carried out under reflux condition for 24 hr. The reaction end was determined and confirmed using thin layer chromatography (TLC). After completion of the reaction, acetonitrile was evaporated under reduced pressure by rotary evaporator apparatus. Then, ethyl acetate / water were added to the residue and the aqueous layer was discarded. The organic phase was washed two times by sodium bicarbonate 5% and also brine. Anhydrous sodium sulfate was applied for drying of the ethyl acetate. The sodium sulfate was removed by filtration and the organic layer was evaporated under reduced pressure. Diethyl ether and *n*-hexane were applied for washing the obtained precipitate.

**Scheme 1:**
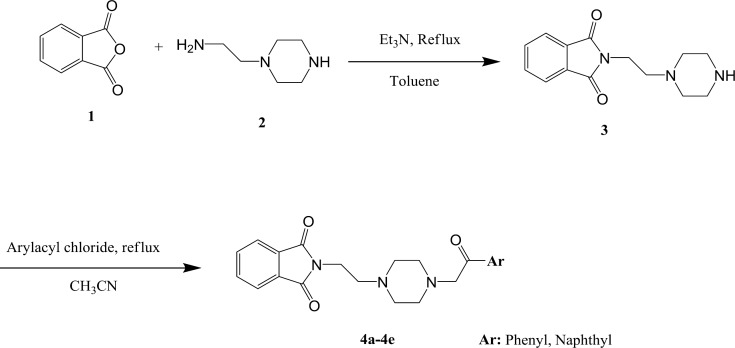
Synthetic pathway for compounds** 4a-4e**

**2-(2-(4-(2-Oxo-2-phenylethyl)** **piperazin-1- yl)ethyl)isoindoline-1,3-dione (4a)**

^1^H NMR (CDCl_3_, 400 MHz) δ (ppm): 1.33 (t, 2H, phthalimide-CH_2_-CH_2_-piperazine), 2.5-3.37 (m, 8H, Piperazine), 3.00 (t, 2H, phthalimide-CH_2_-CH_2_-piperazine), 3.99 (s, 2H, CH_2_CO-Phenyl), 7.22 (m, 5H, Phenyl), 7.46 (m, 2H, Phthalimide), 7.68 (m, 2H, Phthalimide). IR (KBr, cm^-1^) ύ: 3055 (C-H, Stretch, Aromatic), 2927 (C-H, Stretch, Aliphatic), 1712 (C=O, Stretch). MS (*m/z*, %): 378 (M^+^, 70), 272 (100), 257 (10), 231 (12), 217 (12), 203 (10), 174 (20), 160 (15), 125 (15), 105 (40), 98 (80), 77 (30), 56 (25).

**2-(2-(4-(2-(4-Fluorophenyl)-2-oxoethyl)piperazin-1-yl)ethyl)isoindoline-1,3-dione (4b)**

^1^H NMR (CDCl_3_, 400 MHz) δ (ppm): 3.00-4.15 (m, 12H, Aliphatic), 4.34 (s, 2H, CH_2_-CO), 7.21 (m, 4H, 4- Fluorophenyl), 7.48-7.91 (m, 4H, Phthalimide). IR(KBr, cm^-1^) ύ: 3057 (C-H, Stretch, Aromatic), 2924 (C-H, Stretch, Aliphatic), 1712 (C=O, Stretch). MS (*m/z*, %): 396 (30), 272 (80), 260 (45), 174 (15), 123 (60), 99 (100), 76 (15), 56 (25).

**2-(2-(4-(2-(3-Chlorophenyl)-2-oxoethyl)piperazin-1-yl)ethyl)isoindoline-1,3-dione (4c)**

^1^H NMR (CDCl_3_, 400 MHz) δ (ppm): 1.25 (m, 2H, phthalimide-CH_2_-CH_2_-piperazine), 3.04-4.10 (m, 12H, Aliphatic), 7.58 (m, 4H, 3-Chlorophenyl), 7.77 (m, 2H, Phthalimide), 7.99 (m, 2H, Phthalimide). IR (KBr, cm^-1^) ύ: 3074 (C-H, Stretch, Aromatic), 2924 (C-H, Stretch, Aliphatic, Asymmetric), 2854 (C-H, Stretch, Aliphatic, Symmetric), 1708 (C=O, Stretch). MS (*m/z*, %): 413 (M^+^+ 2, 2), 411 (M^+^, 5), 314 (20), 312 (5), 310 (35), 292 (75), 268 (10), 249 (5), 235 (10), 205 45), 139 (100), 75 (55). 

**2-(2-(4-(2-(2,4-Dichlorophenyl)-2-oxoethyl)piperazin-1-yl)ethyl)isoindoline-1,3-dione (4d)**

^1^H NMR (CDCl_3_, 400 MHz) δ (ppm): 1.27 (t, 2H, phthalimide-CH_2_-CH_2_-piperazine), 3.09 (t, 2H, phthalimide-CH_2_-CH_2_-piperazine), 3.51 (m, 4H, Piperazine), 3.82 (m, 6H, Aliphatic), 7.53 (m, 3H, 2,4-Dichlorophenyl), 7.79 (m, 4H, Phthalimide). IR (KBr, cm^-1^) ύ: 3059 (C-H, Stretch, Aromatic), 2927 (C-H, Stretch, Aliphatic), 1708 (C=O, Stretch). MS (*m/z*, %): 446 (M^+^, 2), 432 (5), 414 (5), 364 (5), 301 (10), 288 (25), 272 (80), 260 (100), 217 (12), 203 (20), 174 (80).

**2-(2-(4-(2-(Naphthalen-2-yl)-2-oxoethyl)piperazin-1-yl)ethyl)isoindoline-1,3-dione (4e)**

^1^H NMR (CDCl_3_, 400 MHz) δ (ppm): 1.33 (t, 2H, phthalimide-CH_2_-CH_2_-piperazine), 2.57 (m, 4H, Piperazine), 3.13 (t, 2H, phthalimide-CH_2_-CH_2_-piperazine), 3.75 (m, 6H, Aliphatic), 7.30-8.58 (m, 7H, Naphthyl), 7.78 (m, 2H, Phthalimide), 7.97 (m, 2H, Phthalimide). IR (KBr, cm^-1^) ύ: 3055 (C-H, Stretch, Aromatic), 2931 (C-H, Stretch, Aliphatic), 1708 (C=O, Stretch). MS (*m/z*, %): 427 (M^+^, 10), 272 (95), 203 (10), 174 (25), 155 (40), 127 (75), 99 (100), 76 (15), 56 (50).

***Anti-acetylcholinesterase assay***

Lyophilized powder of acetylcholinesterase from electric eel source (AChE, E.C. 3.1.1.7, Type V-S, 1000 unit) was purchased from Sigma-Aldrich (Steinheim, Germany). 5,5'-Dithiobis-(2-nitrobenzoic acid, DTNB), potassium dihydrogen phosphate (KH_2_PO_4_), dipotassium hydrogen phosphate (K_2_HPO_4_), potassium hydroxide (KOH), sodium hydrogen carbonate (NaHCO3), and acetylthiocholine iodide were purchased from Fluka (Buchs, Switzerland). Spectrophotometric measurements were run on a Cecil BioAquarius CE 7250 Double Beam Spectrophotometer.

**Table 1:** Physicochemical properties of synthesized compounds

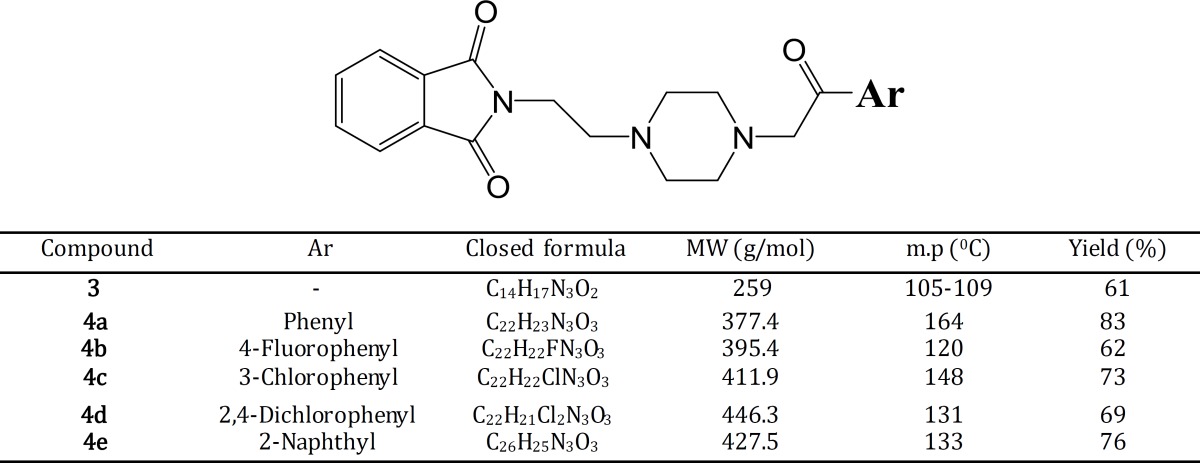

Compounds **4a-4e** were dissolved in a mixture of 20 ml distilled water and 5 ml methanol and then diluted in 0.1 M KH_2_PO_4_/K_2_HPO_4_ buffer (pH 8.0) to yield a final concentration range. According to the literature, the Ellman test was performed for assessment of the anticholinesterase activity of intended compounds *in vitro*. To achieve 20-80% inhibition of AChE activity five different concentrations of each compound were tested. Compounds **4a-4e** were added to the assay solution and preincubated at 25^°^C with the enzyme for 15 min followed by adding 0.075 M of acetylthiocholine iodide. After rapid and immediate mixing the change of absorption was measured at 412 nm. The blank reading contained 3 ml buffer, 200 µl water, 100 µl DTNB and 20 µl substrate. The reaction rates were calculated, and the percent inhibition of test compounds was determined. Each concentration was analyzed in triplicate, and IC_50_ ± SD values were determined graphically from inhibition curves (log inhibitor concentration vs percent of inhibition) (21, 22).

## Results

***Chemistry***

Table 1 shows all compounds **3** and **4a-4e **synthesized with high yield. Compound **3** was prepared with 61% and a range of 62-83% of yields was obtained from final compounds. All synthesized compounds **4a-4e** were afforded as orange powders and compound **3** as yellowish powder. Related melting points were measured and a range of 120-164°C was recorded in this series. All final compounds **4a-4e** rendered a sharp melting point. Whereas, compound **3** exhibited at 105-109°C a ranged melting point.

***Enzymatic assay***

Inhibitory potency of final compounds **4a-4e** was evaluated towards acetylcholinesterase enzyme. Ellman test protocol was applied and the inhibitory potency of tested compounds was reported in Table 2 as IC_50_ ± SD. Donepezil was used as reference drug and it demonstrated a high inhibitory effect (0.41 ± 0.09 µM) towards acetylcholinesterase. A range of 16.42 ± 1.07 µM to 63.03 ± 4.06 µM was obtained for tested derivatives. All of them exerted a lower inhibitory activity than donepezil. 

## Discussion

***Chemistry***

 A new series of donepezil-like analogs based on the phthalimide structure were synthesized. Piperazine moiety was also introduced into these compounds as bioisosteric replacement of the piperidine ring in donepezil. The intermediate compound **3 **was prepared through a Gabriel synthetic procdure of phthalimde. In fact, phthalimic anhydride was treated under reflux condition by *N*-amnioethylpiperazine (**2**) in toluene solvent. The reaction was run for 24 hr and intended product (compound **3**) were obtained and used for synthesis of **4a-4e** derivatives. Final compounds **4a-4e** was obtained in a range of 62-83% yields. The lowest yield was then observed for compound **4b **with 4-fluorophenyl moiety and compound **4a **with phenyl substituent was obtained with highest yield in this series.

^1^H NMR spectra were obtained in CDCl_3_ as deutrated solvent and compared to tetramethylsilane (TMS) as internal standard. IR spectra were recorded using KBr disk and the presence of carbonyl peak was a sign for synthesized compounds. Mass spectra were obtained and the most frequent peaks (fragments) were reported. Fragments with 272 *m/z* and 174 *m/z* were the most probable fragments and were observed in the most cases. Melting points were calculated using melting point analyser on open capillary tubes. Compound **4b** with 4-fluorophenyl moiety showed the lowest melting point at 120°C. Nevertheless, compound **4a** with phenyl substituent exerted the highest melting point at 164°C.

**Table 2:** sults of acetylcholinesterase inhibitory activity of compounds **4a-4e** (IC_50 _, µM)

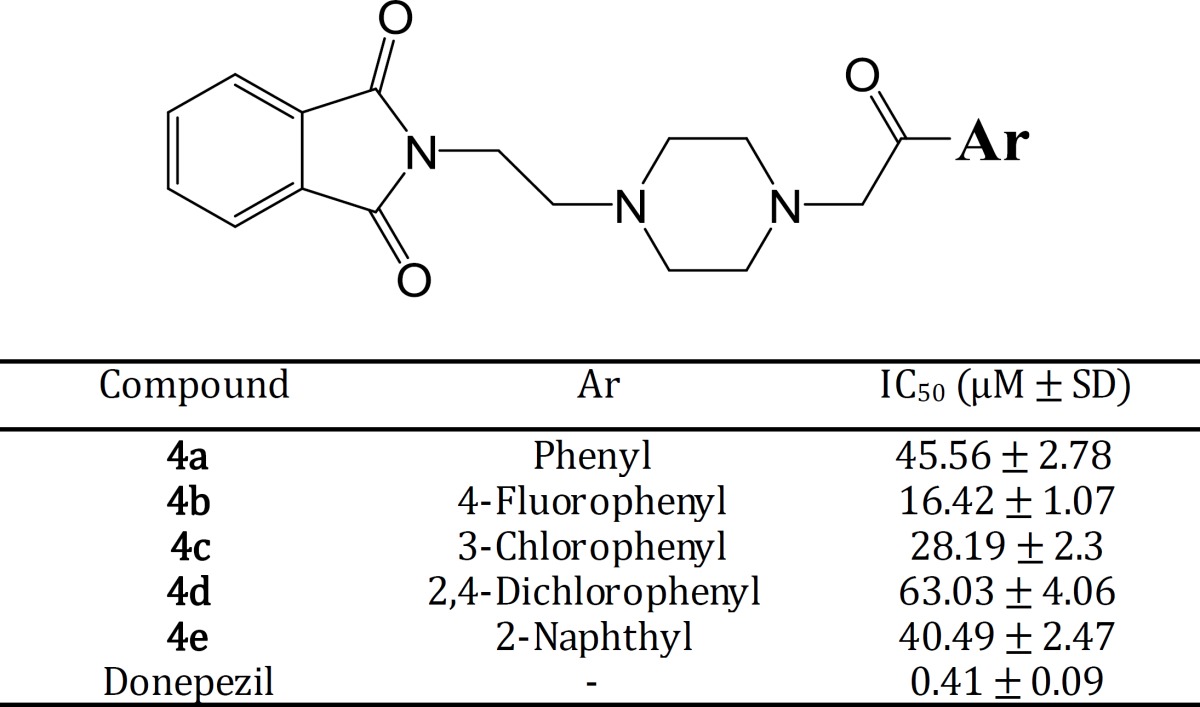

***Enzymatic assay***

 The anti-acetylcholinesterase activity of final compounds **4a-4e** was investigated towards acetylcholinesterase. The related acetylcholinesterase enzyme that applied in this test was with electric eel source and the obtained results (IC_50_) were compared to donepezil as a reference drug. It was shown that, none of the synthesized compounds exerted superior activity in comparison with donepezil. Compound **4b** with 4-fluorophenyl substituent was the best inhibitor (IC_50_ = 16.42 ± 1.07) of acetylcholinesterase enzyme in this series. Whereas, compound **4d** with 2,4-dichlorophenyl group exhibited the lowest inhibitory potency towards acetylcholinesterase. In General, introduction of an electron withdrawing group such as chlorine (compound **4c**) and fluorine (**4b**) enhanced the inhibitory effects of these compounds. More electronegative groups caused more raise in potency. Insertion of chlorine atoms concurrently at positions *ortho* and *para* of the phenyl ring as applied in compound **4d** was so detrimental for activity. It can be proposed that steric hindrance may also be an essential 

factor for ligand-receptor interaction. Fusion of a second phenyl moiety to the phenyl ring (naphthyl group) caused an increase in activity compared to phenyl. However, as mentioned above for compound **4d**, naphthyl moiety also can cause a steric hindrance and eventually decreased the activity. 

## Conclusion

Compounds **4a-4e **were synthesized as new analogs of donepezil based on phthalimide substructure. All of the presented derivatives in this research demonstrated inferior potency than donepezil in Ellman test. Although synthesized compounds rendered low anticholinesterase activity, these derivatives especially compound **4b** could be suggested as potential inhibitors of acetylcholinesterase enzyme. 
